# Ranitidine and finasteride inhibit the synthesis and release of trimethylamine N-oxide and mitigates its cardiovascular and renal damage through modulating gut microbiota

**DOI:** 10.7150/ijbs.40934

**Published:** 2020-01-14

**Authors:** Junfeng Liu, Lingyun Lai, Jiajia Lin, Jiajia Zheng, Xiaoli Nie, Xiaoye Zhu, Jun Xue, Te Liu

**Affiliations:** 1 Division of Nephrology, Huashan Hospital, Fudan University, Shanghai 200040, China; 2 Shanghai Geriatric Institute of Chinese Medicine, Shanghai University of Traditional Chinese Medicine, Shanghai 200031, China

**Keywords:** Trimethylamine N-oxide, gut microbiota, ranitidine, finasteride

## Abstract

Trimethylamine N-oxide (TMAO) leads to the development of cardiovascular and chronic kidney diseases, but there are currently no potent drugs that inhibit the production or toxicity of TMAO. In this study, high-fat diet-fed ApoE-/- mice were treated with finasteride, ranitidine, and andrioe. Subsequently, the distribution and quantity of gut microbiota in the faeces of the mice in each group were analysed using 16S rRNA sequencing of the V3+V4 regions. Pathological examination confirmed that both ranitidine and finasteride reduced atherosclerosis and renal damage in mice. HPLC analysis also indicated that ranitidine and finasteride significantly reduced the synthesis of TMAO and the TMAO precursor delta-Valerobetaine in their livers. The 16S rRNA sequencing showed that all 3 drugs significantly increased the richness and diversity of gut microbiota in the model mice. Bioinformatic analysis revealed that the faeces of mice treated with ranitidine and finasteride, had significant increases in the number of microbes in the families g_Helicobacter, f_Desulfovibrionaceae, *Mucispirillum_schaedleri_*ASF457, and g_Blautia, whereas the relative abundances of microbes in the families *Enterobacter_*sp._IPC1-8 and g_Bacteroides were significantly reduced. The microbiota metabolic pathways, such as nucleotide and cofactor and vitamin metabolism were also significantly increased, whereas the activities of metabolic signalling pathways related to glycan biosynthesis and metabolism and cardiovascular diseases were significantly reduced. Therefore, our study indicates that in addition to their known pharmacological effects, ranitidine and finasteride also exhibit potential cardiovascular and renal protective effects. They inhibit the synthesis and metabolism of TMAO and delay the deposition of lipids and endotoxins through improving the composition of the gut microbiota.

## Introduction

Foods containing choline or trimethylamine structures, such as phosphatidyl choline (PC), L-carnitine, and certain marine fish, can be metabolised into trimellitic anhydride (TMA) gas after digestion and absorption in the gastrointestinal tract. TMA is then rapidly oxidised to TMAO by flavin monooxygenase (FMO3) or other flavin monooxygenases (FMOxs) secreted by the liver. Bennett et al. found that 3 FMOxs were involved in the process of TMA-to-TMAO conversion, of which FMO3 had highest activity 1, 2. Wang et al. previously demonstrated that a high plasma TMAO concentration was associated with atherosclerosis, and plaque size was positively correlated with plasma TMAO concentration 3. Experiments in ApoE-/- mice also confirmed that a diet enriched in L-carnitine could aggravate atherosclerosis and significantly increased TMAO levels in plasma. By comparison, mice fed antibiotics showed significantly reduced TMAO levels and were effectively protected from cardiovascular diseases 4. Many subsequent studies have found that TMAO upregulates macrophage scavenger receptors, leading to macrophage cholesterol accumulation and foam cell formation. This in turn promotes the formation of vascular plaques and inducesa vascular inflammatory response through the p38MAPK and NFκB pathways. TMAO primarily affects cholesterol metabolism and insulin resistance, promotes platelet aggregation, increases thrombosis, induces vascular inflammatory response, and directly causes arterial plaque formation. Tang WH et al. also found that plasma TMAO levels in patients with chronic kidney disease were significantly higher than those in healthy individuals, and that TMAO levels were inversely proportional to long-term survival rates. It has also been shown that a chronic increase in dietary TMAO directly leads to progressive renal fibrosis and dysfunction in animal models 2. The gut microbiota of patients with chronic kidney disease also exhibited obvious dysbiosis, with reduced bacterial diversity and biased community structure. The proportion of the opportunistic γ-Proteobacteria pathogens increased, whereas those of beneficial microbes, such as Roseburia, Coprococcus, and Ruminococcaceae, were significantly reduced 5. Picrust analysis showed that the expression levels of 8 factors related to choline, betaine, L-carnitine, and TMAO metabolism, were altered in patients with chronic kidney disease 5. When the faecal samples of CKD patients were transferred to antibiotic-treated C57BL/6 mice, the plasma TMO levels of the mice significantly increased. Therefore, an imbalance in gut microbiota could increase plasma TMAO levels in patients with CKD and aggravated renal function impairment 5. Furthermore, studies have shown that safe and well-tolerated therapeutic interventions, such as the use of probiotics to improve the gut microbiota, can reduce TMAO levels in the body and mitigate its cardiovascular and renal damage 6-8. Accordingly, investigating methods to reduce TMAO levels in the body will be of great significance in delaying the development of cardiovascular and renal diseases 1, 2.

Wang et al. demonstrated that TMAO production was dependent on the gut microbiota, and that germ-free mice failed to provide the required gut microbiota for TMAO production 3. This was confirmed by Romano et al. who showed that 9 bacterial strains found in the human body could convert choline in food into TMA, suggesting that blood TMAO levels could be lowered by reducing choline intake 9. Other studies have also shown that the amount of TMAO produced in the human body is related to the gut microbial enterotypes, and that gut microbiota containing higher proportions of *Prevotella* are associated with higher TMAO production than gut microbiota containing higher proportions of Bacteroides 10. The gut microbiota also numerous interacts with the human body in numerous other ways, including modulating intestinal development and mucosal barrier function, controlling nutrient uptake and metabolism, promoting immune tissue maturation, and preventing the growth of pathogenic microbes. The gut microbiota also contributes to food digestion through glycolysis or protein hydrolysis. In the glycolytic pathway, the gut microbiota is responsible for the production of short-chain fatty acids, which play a protective and immunomodulatory role 11. During protein hydrolysis, protein fermentation can induce the formation of short-chain fatty acids and the generation of other co-metabolites, such as ammonia, amines, thiols, phenols, and hydrazines. Some of these metabolites are toxic and are potential causative factors of uraemia. Collectively, the gut microbiota plays a fundamental role in systemic immunity and metabolism. In addition, some studies have indicated that drugs such as ranitidine and finasteride are substrates for FMO and can compete with TMA for FMO-binding, reducing TMAO production 12-15. Furthermore, treatment of male rats with the 5α-reductase inhibitor, finasteride, produced a long-lasting effect on depressive-like behaviour, hippocampal neurogenesis, neuroinflammation, and gut microbiota composition 16.

Based on the above evidence, this study aimed to investigate the modulation of the gut microbiota by ranitidine and finasteride,which reduces TMAO synthesis in mice, to examine the protective effects of these drugs against cardiovascular and renal damage.

## Materials and Methods

### Mouse model groups and drug interventions

A total of 32 male, SPF-grade, 6-to-8 week old, ApoE-/- C57/BABL mice weighing 30±5g were purchased from Shanghai Model Organisms Company (Shanghai, China). License number: SCXK (Shanghai) 2014-0002. After 1 week of adaptive feeding, the ApoE-/- C57/BABL mice were randomly divided into 4 groups: (1) The model control group (fed a high-fat diet + equal volume of saline); (2) The ranitidine group (fed a high-fat diet + ranitidine at 1.5 mg/30g body weight); (3) The andrioe group (fed a high-fat diet + andrioe at 0.2 mg/30g body weight); (4) The finasteride group (fed a high-fat diet + finasteride at 1.5 mg / 30g body weight). Each group consisted of 8 rats. Intervention was given once a day for 4 consecutive weeks. This study was approved by the ethics committee of the Shanghai Geriatric Institute of Chinese Medicine (SHAGESYDW201608). All experiments conformed to the experimental animal regulations of the Ministry of Science and Technology.

### Haematoxylin-eosin (H&E) staining

H&E staining was used to observe the pathologic histomorphology of the mice's aortas. The aortaswere fixed in 10% formaldehyde (Beyotime Biotechnology, HangZhou, China), and the aortic arch located 0.5 cm from the aorta root was excised. The aortic arch was subjected to routine dehydration and embedded in paraffin. Serial sections (5 μm) were prepared starting from the aorta root. The sections were stained with H&E and observed under a light microscope.

### MASSON staining

Sections with plaques at the aortic root were selected for deparaffinisation. The sections were washed with double-distilled water for 5 min and then stained with haematoxylin (Beyotime Biotechnology) for 5-10 min, followed by thorough rinses with water. The sections were subsequently counterstained with Masson's Ponceau Acid Fuchsin solution (Beyotime Biotechnology) for 6-10 min, and then rinsed in 2% ice-cold aqueous acetic acid (Beyotime Biotechnology) for 5 s. The sections were then differentiated for 3-5 min with 1% aqueous phosphomolybdic acid (Beyotime Biotechnology), stained by direct immersion in aniline blue for 5 min and then washed with 0.2% aqueous glacial acetic acid (Beyotime Biotechnology) for several seconds. The stained sections were cleared, sealed, and photographed.

### Lipid profile

Mouse peripheral blood was collected and left to stand at 4°C for 4 h. The blood wasthen centrifuged at 10,000r/min for 10 min at 4°C, and the supernatant was collected. TC, TG, HDL-C, and LDL-C (Nanjing Jiancheng Bioengineering Institute, Nanjing, China) in serum were detected with a kit according to the manufacturer'sdirections.

### Real-time quantitative PCR (qPCR)

Total RNA was extracted from cells from each group with Trizol Reagent (Invitrogen Life Technologies, Carlsbad, CA, USA) according to the manufacturer's protocol. The total RNA was treated with Dnase I (Sigma-Aldrich, St Louis, MO, USA), quantified, and reverse transcribed into cDNA using a ReverTra Ace-α First Strand cDNA Synthesis Kit (Toyobo (Shanghai) Biotech Co., Ltd., Shanghai, China). qRT-PCR was performed using a RealPlex4 real-time PCR detection system from Eppendorf Co. LTD (Germany) With SyBR Green RealTime PCR Master MIX (TOYOBO). The qRT-PCR protocol consisted of 40 amplification cycles with the following conditions: denaturation at 95°C for 15s; annealing at 58°C for 30s; and extension at 72°C for 42s. The relative expression levels of the genes were determined using the 2^-ΔΔCt^ calculation method, where ΔCt = Ct_genes-Ct_18sRNA; ΔΔCt = ΔCt_all_groups-ΔCt_blank_control_group. The levels of mRNA expression were normalised to the expression of the 18s rRNA. The primers used for amplification of each gene were: Fmo3-F: GGCCTGTGGAAATTCTCAGAC; Fmo3-R: AAGTCATCGGGATAGGGGAAG; 18s rRNA-F: GGAGAAACCTGCCAAGTATGA; 18s rRNA-R: CAACCTGGTCCTCAGTGTAGC.

### Analysis by HPLC-ELSD

The analyses were performed by HPLC-ELSD with an shimadzu LC20 series liquid chromatograph using a Diamonsil C18 column, 50×4.6 mm, particle size 5 μm. The chromatography was conducted isocratically with mobile phase of methanol-0.1% formic acid in water (5-95) at flow rate of 300 μL/min.Volumes of 10 μL of standard solution or sample were injected. Compounds were identified on the basis of their retention times. Quantification of each substance was generally obtained by comparison of the peak area with the respective calibration curve built with standard solutions. The concentrations of each compound were determined by comparison with the relative calibration curve. Standard stock solution of TMAO (Sigma-Aldrich Chemical) was prepared at 100 mg/L. Additional calibration levels (5, 2, 1, 0.5, 0.3, 0.2 and 0.1 mg/L) were prepared by serial dilution with methanol. Standard stock solution of Delta-Valerobetaine (Sigma-Aldrich Chemical) was prepared at 10 mg/L. Additional calibration levels (5, 3, 1, 0.5 and 0.1 mg/L) were prepared by serial dilution with methanol. The calibration curves were build using these standard solutions. The linear regression analysis was carried out by plotting the peak areas versus the concentrations of the tmao standard solutions. The linear regression analysis was carried out by the logarithm plotting the peak areas versus the concentrations of the tmao standard solutions.The linearity of the instrumental response was assessed by correlation coefficients (r^2^) > 0.99 for all analytes. Each sample was analyzed in triplicate and the mean concentration value of each compound was calculated.

### Gut microbiota analysis

According to the previous study 17, 18, fresh fecal samples were collected during the final 5 days for the gut microbial analysis. Bacterial genomic DNA was extracted from frozen samples stored at -80°C. The V3 and V4 regions of the 16S rRNA gene comprising were amplified by PCR using specific bacterial primers (F primer: 5'-ACTCCTACGGGAGGCAGCA-3'; R primer: 5'-GGACTACHVGGGTWTCTAAT-3'). High-throughput pyrosequencing of the PCR products was performed on an Illumina MiSeq platform at Biomarker Technologies Co. Ltd. (China). The raw paired-end reads from the original DNA fragments were merged using FLASH32 and assigned to each sample, according to the unique barcodes. QIIME 19 (version 1.8.0) UCLUST 20 software was used based on 97% sequence similarity. The tags were clustered into OTUs. The alpha diversity index was evaluated using Mothur software (version, v.1.30). To compare the diversity index among samples, the number of sequences contained in each sample was standardized. Analysis treasure included OTU rank, rarefaction, and Shannon curves, and the Shannon, Chao1, Simpson, and ACE indexes were calculated. For beta diversity analysis, heatmaps of RDA-identified key OTUs, PcoA 21, NMDS 22, and UPGMA were obtained using QIIME. The LDA-effect size (LEfSe) method was used for the quantitative analysis of biomarkers in each group. Briefly, LEfSe analysis, an LDA threshold >4, the non-parametric factorial Kruskal-Wallis sum-rank test, and the unpaired Wilcoxon rank-sum test were performed to identify the most differently abundant taxa 23, 24.

### Western blotting analysis

Total proteins extracts of each group cells were resolved by 12% SDS-PAGE and transferred on PVDF (Millipore, Bedford, MA, USA) membranes. After blocking, the PVDF membranes were washed 1 times for 15 min with TBST (Beyotime Biotechnology) at room temperature and incubated with primary antibody (rabbit anti-mouse FMO (1:1000, Cell Signaling Technology, MA, USA)) at 4°C overnight. After washing 4 times for 15 min with TBST at room temperature once more, membranes were incubated with secondary antibody peroxidase-linked goat anti-rabbit IgG (1:1000, Cell Signaling Technology, MA, USA) at room temperature for 45 min. After washing 4 times for 15min with TBST at room temperature once more, the immunoreactivity was visualized by enhanced chemiluminescence (ECL kit, Beyotime Biotechnology), and membranes were exposed to Kodak XAR-5 films (Sigma-Aldrich Chemical).

### Statistical analysis

Each experiment was performed as least three times and data were shown as the mean ± SD. The differences were evaluated using Student's *t*-tests. The probability of < *0.05* was considered to be statistically significant.

## Results

### 1. Both ranitidine and finasteride reduced atherosclerosis and renal damage in mice

**Figure 1:**
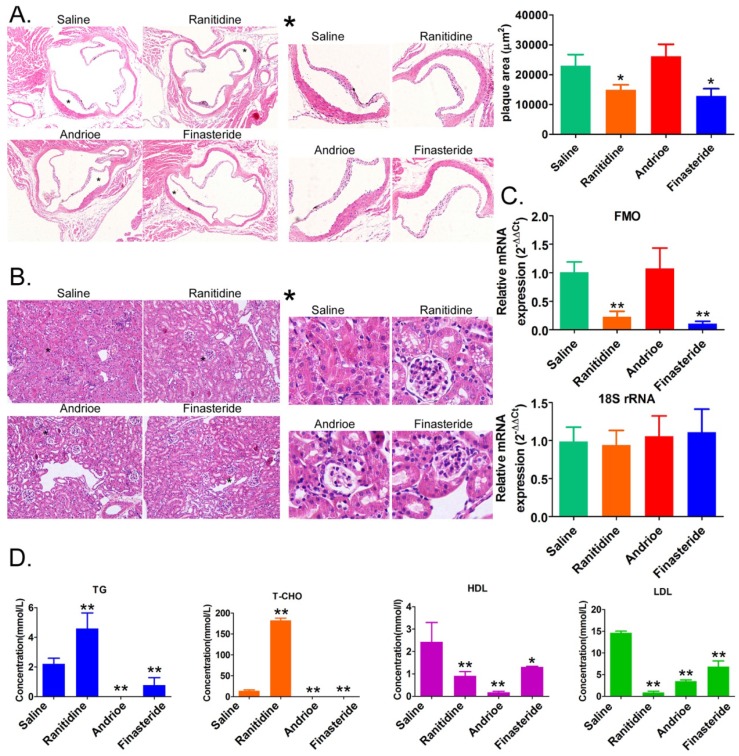
Both ranitidine and finasteride reduced atherosclerosis and renal damage in mice. (A) Representative H&E stains of the aortic arches of mice from each group. Magnification × 100. * High-power field. Magnification × 100. (B) H&E stains of the renal tissues of mice in each group. Magnification × 100. * High-power field. Magnification × 100. (C) Cholesterol, triglyceride, high-density lipoprotein, and low-density lipoprotein concentrations in the peripheral blood of mice from each group. * p<0.05 vs Saline group; t test. n=8. (D) qPCR analysis of FMO3 mRNA expression in the liver tissue of mice from each group. * p<0.05 vs Saline group; t test; n=8.

H&E staining showed that the aortic roots of mice in the saline-treated group were significantly thickened, and the plaque sizes were much larger than those in the ranitidine and finasteride-treated groups (Figure 1A). The aortic roots of the mice in the Andrioe-treated group were also thickened, but the thickness was not different from that seen in the saline group (Figure 1A). In the renal tissue of the saline-treated group, the mesangial cells in some regions of the mesangium in the glomeruli exhibited a moderate-to-high degree of proliferation. The renal tubular epithelial cells showed vacuolar degeneration, and some were detached, exposing the naked basement membrane (Figure 1B). In the kidneys of rats treated with ranitidine and finasteride, most of the renal tubular epithelial cells did not show significant damage or detachment. There was no notable infiltration of inflammatory cells in the renal stroma and the mesangial cells in some regions of the mesangium in the glomeruli exhibited a mild degree of proliferation, and the capillary lumen were open (Figure 1B). qPCR and Western blot showed that FMO3 expression in the liver tissue of mice treated with ranitidine and finasteride was significantly lower than in the saline-control and Andrioe-treated groups (Figure 1C, Supplementary figure 1). The lipid profile of the peripheral blood of the mice in each group showed that ranitidine and finasteride significantly reduced the levels of cholesterol, triglyceride, and low-density lipoprotein (Figure 1D). These results indicated that both ranitidine and finasteride reduced atherosclerosis and renal damage in mice.

### 2. Both ranitidine and finasteride reduced TMAO and the TMAO precursor levels in the livers of mice

**Figure 2:**
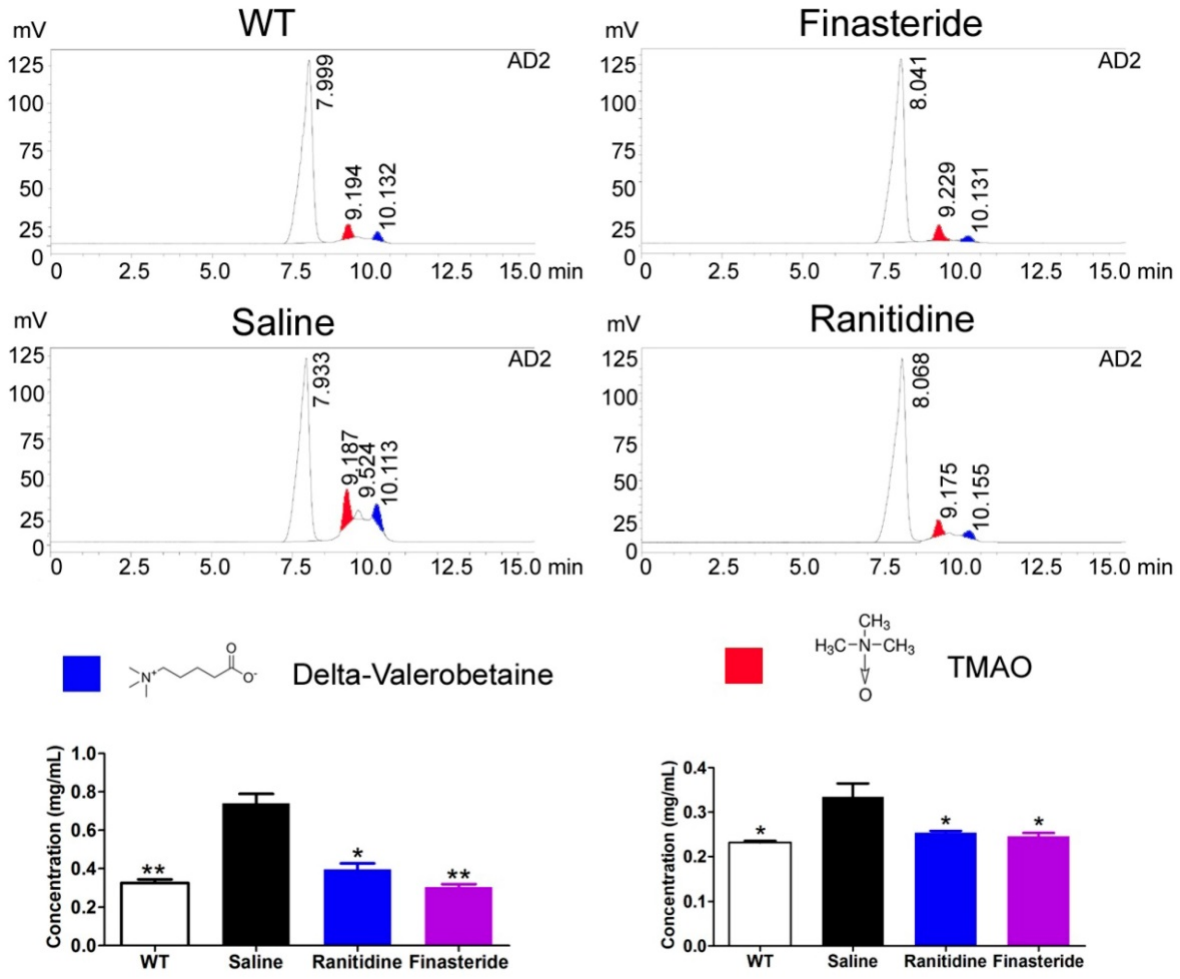
Both ranitidine and finasteride reduced the levels of TMAO and the TMAO precursor in the livers of mice. The levels of the TMAO precursor (delta-Valerobetaine) and TMAO in the livers of mice in each group were determined using HPLC. ** p<0.01 vs Saline group; * p<0.05 vs Saline group; t test; n=8.

As TMAO canlead to atherosclerosis and renal damage, we sought to examine the levels of the TMAO precursor (delta-Valerobetaine) and TMAO in the livers of the mice in each group using HPLC. ApoE-/- mice fed a normal diet had low levels of these substances in their livers (Figure 2). In contrast, the high-fat diet significantly increased the levels of delta-Valerobetaine and TMAO in the livers of ApoE-/- mice (Figure 2). Treatment with either ranitidine or finasteride reduced the levels of delta-Valerobetaine and TMAO in the livers of high-fat diet-fed ApoE-/- mice (Figure 2).

### 3. Both ranitidine and finasteride altered the distribution of gut microbes in mice

**Figure 3:**
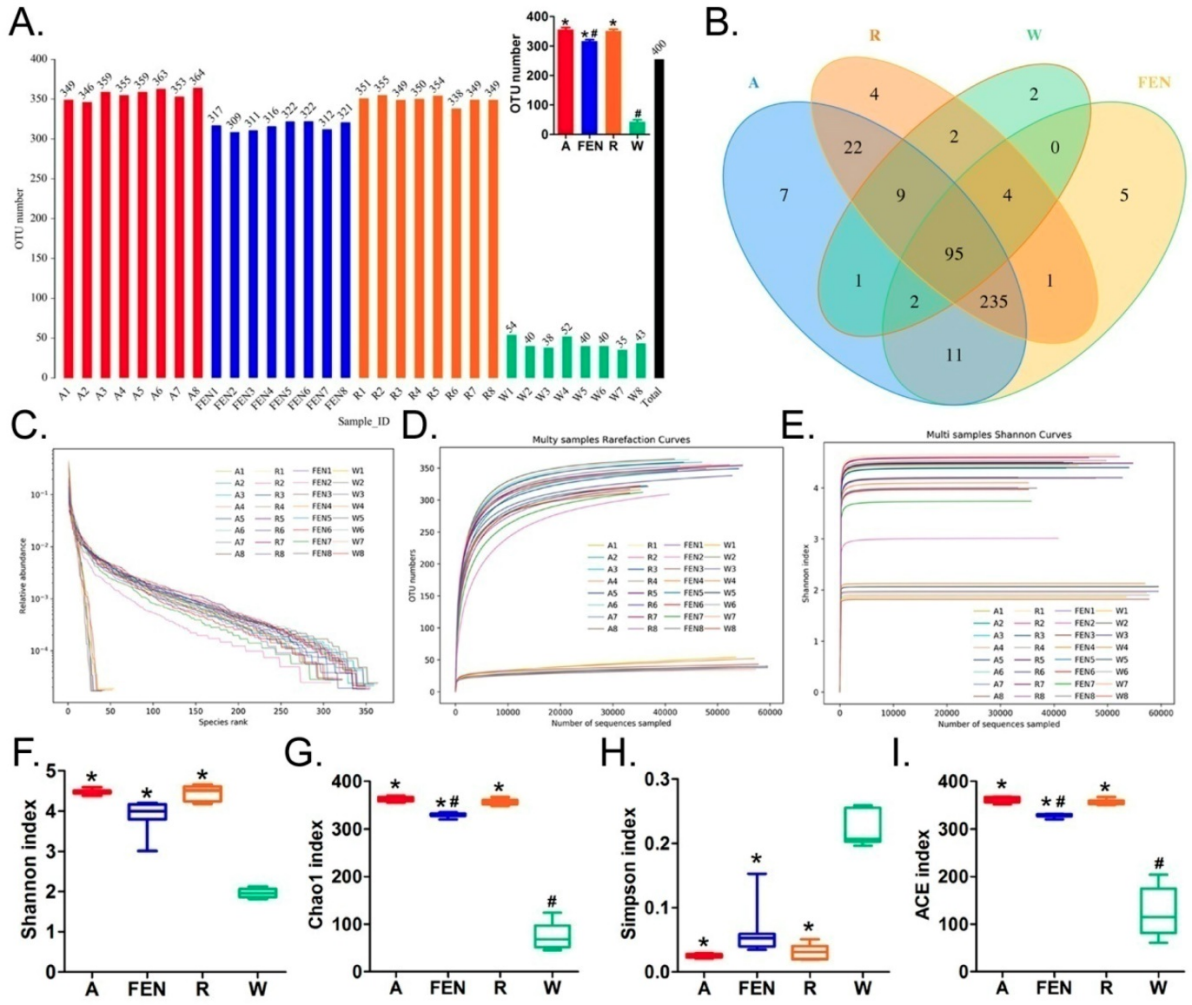
Distribution of gut microbes from each group of mice. (A) Distribution of the number of OTUs in each sample. * p<0.05 vs W group; t test; n=8. ^#^ p<0.05 vs A group; t test; n=8. (B) Venn diagram of the OTUs in each sample. (C) Rank Abundance Curve (D) Rarefaction Curve (E) Shannon Index (F) (G) (H) and (I) Alpha diversity indices of OTU distribution in each sample. * p<0.05 vs W group; t test; n=8. ^#^ p<0.05 vs A group; t test; n=8.

The faeces of the mice in the model group (W), Andrioe-treated group (A), Ranitidine-treated group (R) and Finasteride-treated group (FEN), were collected, and their bacteria were subjected to 16S rRNA sequencing of the v3+ v4 region to assess the composition of the gut microbiota and the distribution of specific microbes. A total of 2,559,179 paired-end reads were obtained from the 32 samples sequenced. The paired-end reads were merged. After filtering, a total of 1,911,075 clean reads were generated with at least 55,290 clean reads per sample. The average number of clean reads per sample was 59,721 (Table S1). Using QIIME (version 1.8.0) UCLUST software set to 97% sequence similarity, the sequences were clustered into operational taxonomic units (OTUs). The number of OTUs in the R, FEN, and A groups were significantly different than the W group (there were significantly more OTUs in the former 3 groups). However, there were significantly fewer OTUs in the FEN group than the A group, while there was no statistically significant difference between the R and A groups (Figure 3A-E). We subsequently generated a Venn diagram of the OTUs (Figure 3B), as well as the OTU rank, Rarefaction curves, Shannon index curves, Chao1 curves, Simpson curves, and ACE curves (Figure 3F-I; Table S2). Consistent with the change in the number of OTUs, interventions with FEN, R, and A, significantly increased the richness and diversity of the gut microbiota in high-fat diet-fed ApoE-/- mice.

**Figure 4:**
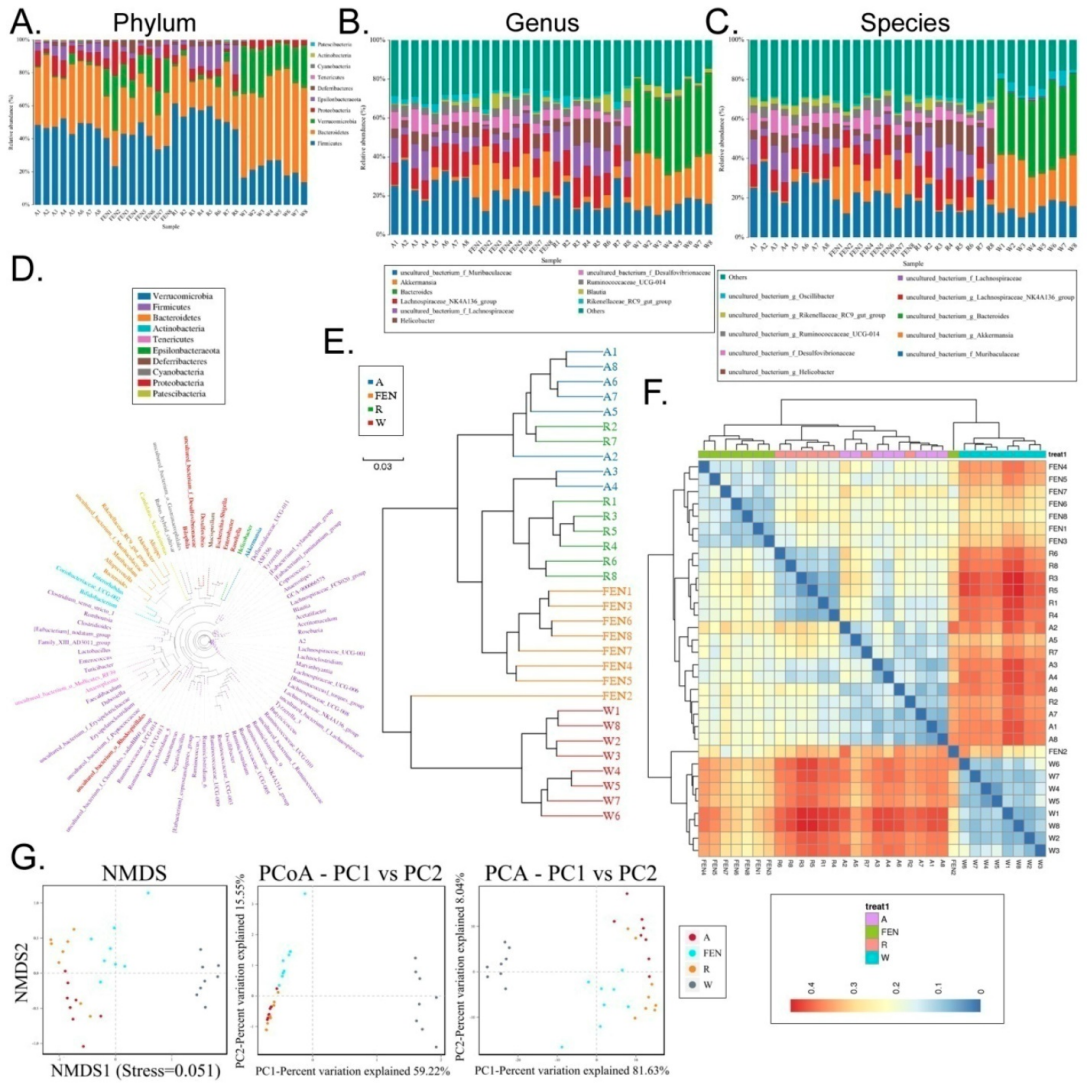
Taxonomic distribution of gut microbiota in each group of mice. Taxonomic distribution of gut microbiota in the mice of each group at the (A) phylum, (B) genus, (C) and species levels. (D) Phylogenetic tree based on the OTUs in each sample at the genus level. (E) Hierarchical clustering analysis of the OTUs in each sample using the unweighted pair-group method with arithmetic mean (F) Heat map analysis on the gut microbiota of mice in each group (G) Beta diversity analysis of the OTU distribution in each sample.

**Figure 5:**
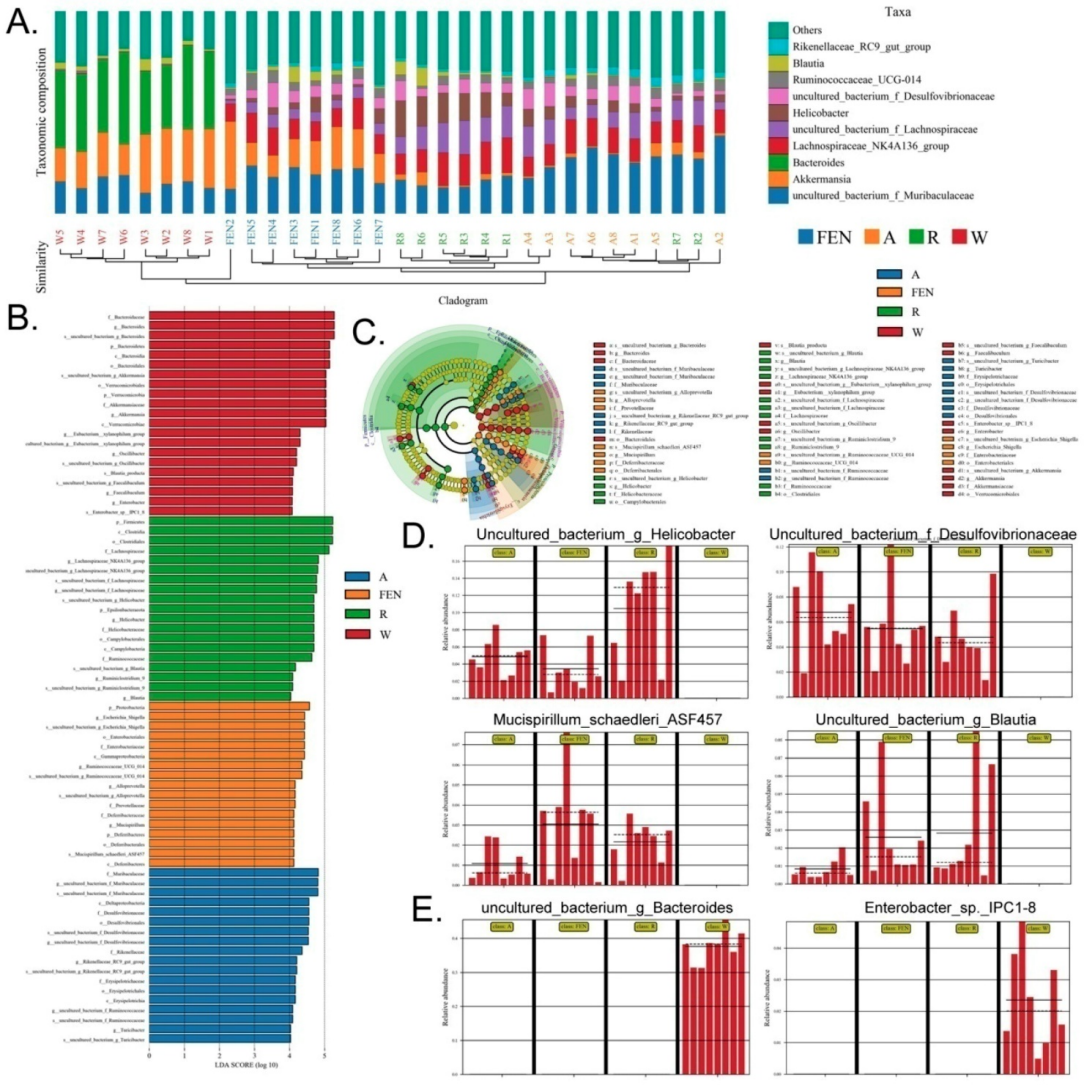
Ranitidine and finasteride alter specific populations of gut microbes in mice. (A) Combined UPGMA clustering tree and histograms of the gut microbiota of the mice in each group (B). (C) Line discriminant analysis (LDA) effect size analysis of the gut microbiota in each sample. (D) Distribution of microbes increased most significantly in the gut microbiota of drug-treated mice (E) Distribution of microbes decreased most significantly in the gut microbiota of drug-treated mice.

Each OTU was assigned to a taxon by comparing the sequence of the OTU with a microbial sequence database allowing the community composition of each sample to be determined. The QIIME software was used to generate the abundance tables for each taxonomic group at the different classification levels (phylum, class, order, family, genus, and species) (Figure 4A-C; Table S3). The community members from each sample at different classification levels were mapped using the R programming language. Analysis at the phylum level showed that the relative abundance of microbes in the phylum Bacteroidia was significantly increased in the gut of mice in the W group, while the relative abundance of Firmicutes was significantly reduced (Figure 4A). Conversely, in the groups of mice treated with FEN and R, the relative abundance of microbes belonging to the phylum Bacteroidia was significantly reduced, but the relative abundance of Firmicutes increased significantly. Further analyses of the microbial differences were conducted at the genus and species levels. Analysis at the genus level showed that compared with the W group, the relative abundance of microbes belonging to the Lachnospiraceae_NK4A136_group was significantly increased in the gut of mice in the FEN group, while the relative abundance of Bacteroides was significantly reduced. Compared with the W group, the mice in the R group showed significant increases in the relative abundances of gut microbes belonging to the Lachnospiraceae_NK4A136_group and uncultured_f_Lachnospiraceae, whereas the relative abundances of Bacteroides and Akkermansia were significantly reduced (Figure 4B). Finally, we analysed the classification of the differentially-distributed microbes. Compared with the W group, the mice in the FEN and R groups showed significant increases in the relative abundances of bacteria from the g_Lachnospiraceae_NK4A136_group, f_Lachnospiraceae, and f_Desulfovibrionaceae in the gut, whereas the relative abundances of g_Bacteroides and g_Akkermansia were significantly reduced (Figure 4C). Cluster analysis showed that the diversity of gut microbes in the mice of each group was mostly attributable to microbes from the phylum Firmicutes (Figure 4D). Meanwhile, hierarchical cluster analysis byunweighted pair-group method with arithmetic mean (UPGMA) suggested that the gut microbiota of the mice in the FEN-treated and the R-treated groups had higher levels of homology and closer genetic backgrounds (Figure 4E-F). Next, the Bray-Curtis algorithm with principal coordinates analysis (PCOA), principal component analysis (PCA), and non-metric multi-dimensional scaling (NMDS) was used to analyse the intergroup differences of the microbiota (Figure 4G). The analyses showed that the population distributions of the microbial communities in the 4 groups of mice were significantly different (Figure 4G). Specifically, the microbes from the W group belonged to a single community, while the microbial communities in the FEN-treated and R-treated groups exhibited similar population distributions and structures. A diagram based on the UPGMA clustering tree and histograms is shown as Figure 5. Based on this comprehensive analysis, we found that Bacteroides was the predominant species in the W group, while the 3 genera Helicobacter, Lachnospiraceae_NK4A136_group, and Ruminococcaceae_UCG-014, were absent (Figure 5A-B). In contrast, Bacteroides was almost absentfrom the FEN-treated and R-treated groups. Rather, the levels of the Lachnospiraceae_NK4A136_group and Helicobacter were increased (Figure 5A-B). These results corroborated the species abundance tables generated using the QIIME software.

Furthermore, high-dimensional biomarkers in the gut microbiota of mice in each group were identified using the line discriminant analysis (LDA) effect size (LEfSe) method (Figure 5C). The threshold LDA score was set to 4.0, and taxa with LDA scores greater than 4 were considered important biomarkers. As shown by the cladogram analysis and the LDA score distribution, the gut microbiota of the FEN-treated and R-treated groups showed significant increases in the number of microbes belonging to families g_Helicobacter, f_Desulfovibrionaceae, *Mucispirillum_schaedleri_*ASF457, and g_Blautia (Figure 5D). In contrast, microbes belonging to the families of *Enterobacter_*sp._IPC1-8 and g_Bacteroides were almost absent in the gut microbiota of the FEN-treated and R-treated groups but were the predominant species specific to the W group (Figure 5E).

### 4. Both ranitidine and finasteride alter the differential expression and metabolic and signalling pathways in the gut microbiota of mice

**Figure 6:**
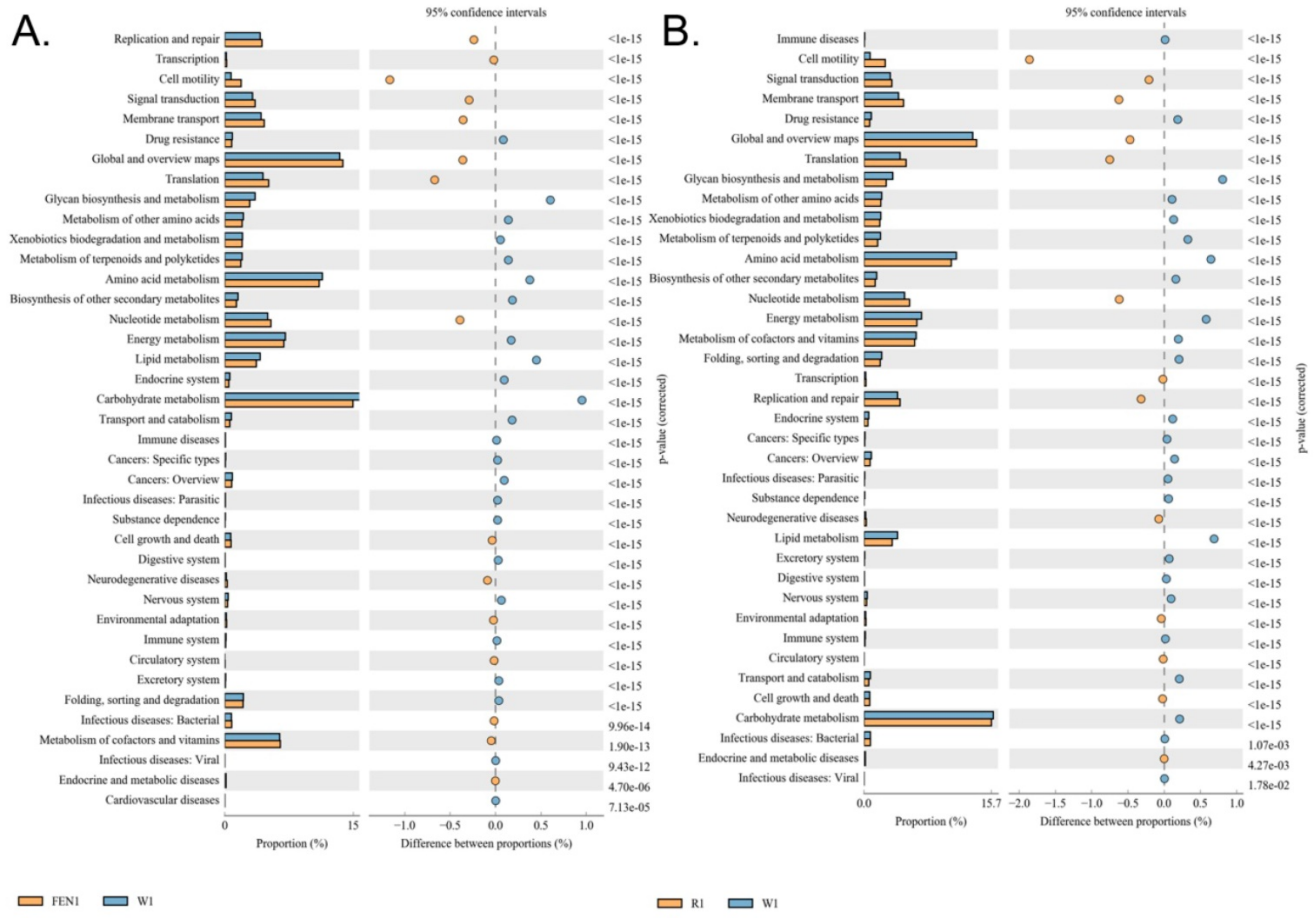
Functional prediction analysis using KEGG pathways based on the 16S sequencing results. (A) Comparison of the finasteride and control groups (B) Comparison of the ranitidine and control groups

The differences in the metabolic pathways of the microbial communities from the different groups were detected by differential analysis using KEGG metabolic pathways, which in turn allowed us to investigate changes in metabolic capability due to adaptation to the environment in the different samples (Figure 6A-B). The analysis showed that compared with the W group, metabolic pathways for substances in the circulatory system, including nucleotide and cofactor and vitamin metabolism were more prevalent in microbes in the mice of the FEN-treated group, while glycan biosynthesis and metabolism was less prevalent (Figure 6A). This could promote cell motility and reduce the activity of the metabolic signalling pathway associated with cardiovascular diseases (Table S4). Comparison of the R-treated and W groups showed that the abundances of metabolic pathways in the R-treated group were highly similar to those in the FEN-treated group (Figure 6B). Similarly, the gut microbes in the mice from the R-treated group could increase the metabolic pathways for substances in the circulatory system, including nucleotide metabolism, and carbohydrate transport and metabolism, while reducing the glycan biosynthesis and metabolism pathway (Figure 6B). This could promote activation cell motility and reduce the activities of the metabolic signalling pathways associated with cardiovascular and infectious (parasitic) diseases (Table S5).

**Figure 7:**
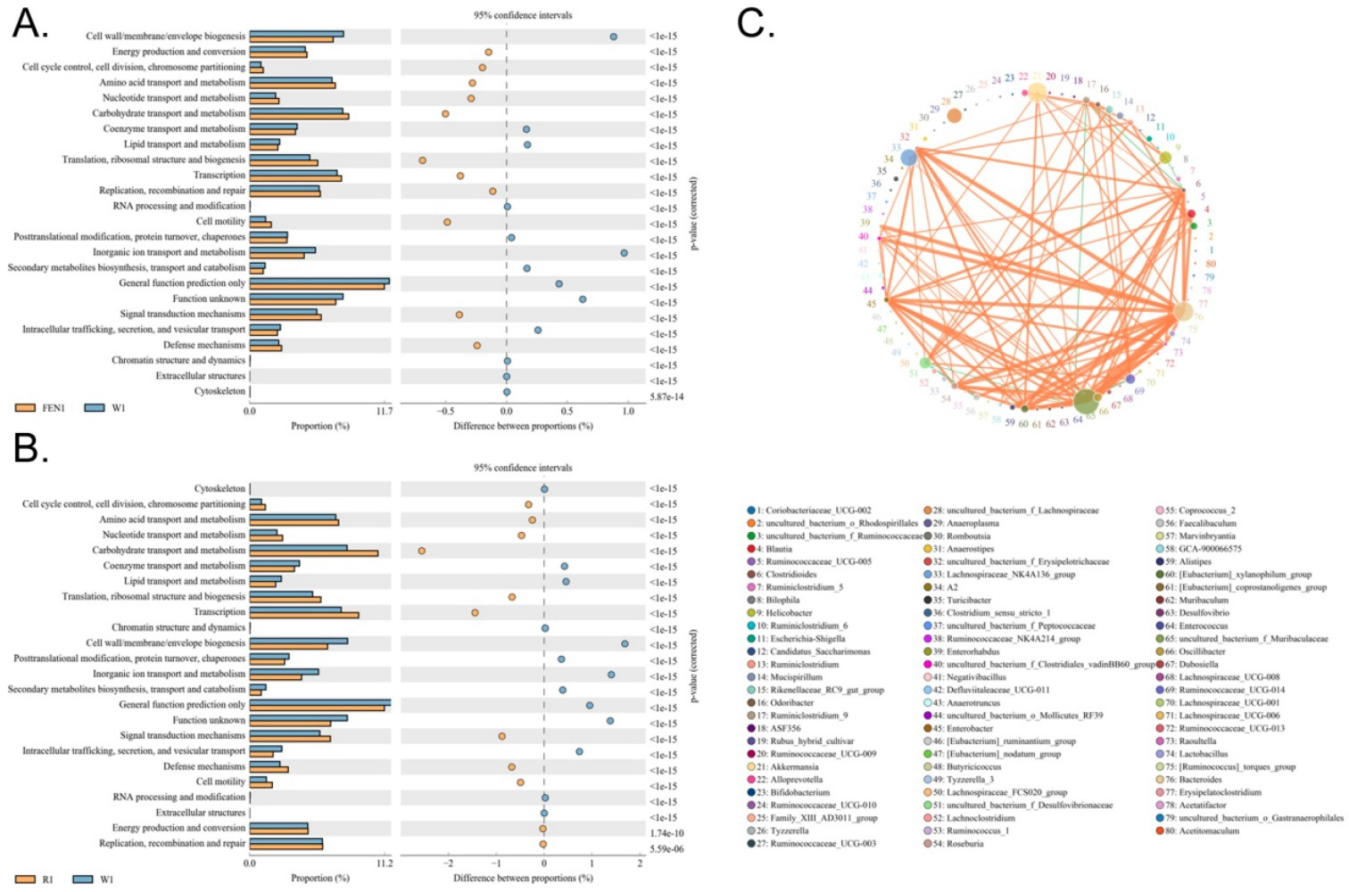
Functional prediction analysis using clusters of orthologous groups of proteins (COG) based on the 16S sequencing results. (A) Comparison of the finasteride and control groups (B) Comparison of the ranitidine and control groups (C) Correlation analysis using the Sparcc algorithm.

Clusters of Orthologous Groups of proteins (COG) could be used to determine the distribution and abundance of homologous protein clusters in the microbiota (Figure 7A-C). Compared with the W group, the gut microbiota of mice in the FEN-treated group showed significant increases in the number of nucleotide transport and metabolism genes, which arerelated to metabolism, and in cell motility (Figure 7A). In contrast, genes related to inorganic ion transport and metabolism were significantly reduced (Table S6). Compared with the W group, the gut microbiota of mice in the R-treated group showed significant increases in genes for nucleotide transport and metabolism and carbohydrate transport and metabolism (Figure 7B). Genes related to cell motility were also significantly increased (Table S7).

## Discussion

Hazen et al. and Hodge et al. utilised metabolomics to predict cardiovascular disease risk and found that 3 phosphatidylcholine metabolites, including choline and TMAO, were associated with increased risk of atherosclerosis in humans and promoted atherosclerosis in mice. Their study suggested that both gut microbial metabolites, TMA and TMAO, promoted the development of atherosclerosis in mice and humans 3, 25, 26. The production of TMAO relies on metabolism by the gut microbiota and the catalysis of specific enzymes in the liver. Therefore, gut microbiota and hepatic FMO are potential targets for drug discovery. Pharmacologically, ranitidine and finasteride should not interact. The former is a potent antagonist of the histamine H2 receptor that can effectively inhibit gastric acid secretion induced by histamine, pentagastrin, and carbachol. It can reduce gastric acid and gastric enzyme activities and is primarily used to treat hyperacidity and heartburn. The latter is a 4-azaindole compound. It is a specific inhibitor of intracellular type II 5α-reductase, which is involved in testosterone metabolism during the formation of the more potent dihydrotestosterone. Finasteride inhibits the conversion of testosterone to dihydrotestosterone, which reduces the size of the prostate, thereby improving symptoms, increasing urine flow rate, and preventing the progression of benign prostatic hyperplasia. None of the above drugs have been previously used in the treatment of atherosclerosis. However, we identified some reports that showed drugs such as ranitidine and finasteride were substrates for FMO and could compete with TMA for FMO-binding, leading to reduced TMAO production 12-15. Furthermore, treatment of male rats with the 5α-reductase inhibitor finasteride produced a long-lasting effect on depressive-like behaviour, hippocampal neurogenesis, neuroinflammation, and gut microbiota composition 16. These studies inspired us tohypothesize that ranitidine and finasteride could indeed improve the synthesis of TMAO. In such case, they were likely to exert a regulatory effect on the ecological distribution of the gut microbiota 3, 25, 26.

In this study, high-fat diet-fed ApoE-/- mice were treated with ranitidine and finasteride. Subsequent pathological analyses and lipid profile for 4 lipids showed that both ranitidine and finasteride significantly lowered cholesterol and triglyceride levels and reduced vascular plaque deposits and renal damage. These results illustrated the modulatory effects of the two drugs on lipids and their potential therapeutic effects on atherosclerosis and renal damage. In addition, HPLC analysis suggested that ranitidine and finasteride also reduced the levels of TMAO and the TMAO precursor in the liver. These results strongly suggested that ranitidine and finasteride could modulate blood lipids through the targeted regulation of TMAO levels which led us to suspect that the gut microbiota was the most direct target of ranitidine and finasteride.

High-throughput sequencing analysis of the 16S gene in the microbial genome revealed that there were significant increases in the quantities of microbes in the families g_Helicobacter, f_Desulfovibrionaceae, *Mucispirillum_schaedleri_*ASF457, and g_Blautia in the faeces of mice treated with ranitidine and finasteride, whereas the relative abundances of microbes in the families *Enterobacter_*sp_.IPC1-8 and g_Bacteroides were significantly reduced. The prevalence of corresponding metabolic pathways, such as nucleotide and cofactor and vitamin metabolismwere significantly increased, whereas those of metabolic signalling pathways related to glycan biosynthesis and metabolism and cardiovascular diseases were significantly reduced.It is known that Blautia can proliferate under the stimulation of prebiotics and is a typical beneficial bacteria 27, 28. Serrano-Villar S et al. found that Blautia was significantly increased in the gut microbiota when normal individuals consumed probiotics 27. Shin JH et al. reported that plasma TMAO levels were significantly lower in subjects who consumed a traditional Korean Diet compared with those who consumed a high-sugar and high-fat American Diet, and the number of Blautia in the gut microbiota in the former group also increased 28. In addition, Sun J et al. reported that IgA-bound bacteria could mitigate high-fat-dietinduced intestinal mucosal barrier damage and lower blood lipids in mice, mainly by stimulating an increase in Desulfovibrionaceae in the host gut microbiota^29^. This showed that Desulfovibrionaceae had positive effects on lowering blood lipids and repairing intestinal barrier function. Meanwhile, *Enterobacter* and *Bacteroides* are typical pathogenic bacteria 30-36. Enterobacter is widely found in nature and is one of the normal bacterial strains found in the gut, but it can also act as an opportunistic pathogen. *Enterobacter_*sp._IPC1-8 can produce extended-spectrum β-lactamase and Amp C enzymes, resulting in the rapid acquisition of antibiotic resistance, particularly towards β-lactam antibiotics 30-33. It can also cause bacterial infections that often involve multiple organ systems; including skin and soft tissue infections, urinary tract infections, and sepsis 30-33. *Bacteroides* are Gram-negative, non-sporulating, obligate anaerobic small bacilli 34-36. They are natural colonizers of the gut, oral cavity, upper respiratory tract, and reproductive tract of humans and animals 34-36. They are predominantly found in the intestinal tract and are 100 to 1000 times more common than *E. coli* in the intestine. They are commonly found in clinical specimens from appendicitis, and sepsis. *Bacteroides* can cause endogenous infections under conditions such as immune dysfunction or dysbiosis due to the long-term use of broad-spectrum antibiotics, hormones, and immunosuppressive agents 34-36. We found that ranitidine and finasteride significantly reduced the abnormal increase in the quantities of *Enterobacter_*sp._IPC1-8 and g_Bacteroides in the gut of mice fed a high-fat diet, suggesting that these 2 drugs could significantly reduce the risk of infection. They also maintained the intestinal mucosal barrier and mucosal immune function and exerted a significant protective effect on the gut. In addition, it was previously reported that the gut microbiota of patients with chronic kidney disease exhibited obvious dysbiosis, in which the proportion of γ-Proteobacteria opportunistic pathogens increased, while the proportion of beneficial microbes, such as *Roseburia*, *Coprococcus*, and Ruminococcaceae, were significantly reduced 5. Showing that changes in the gut microbial communities can indeed affect cardiovascular and renal conditions.

**Figure 8:**
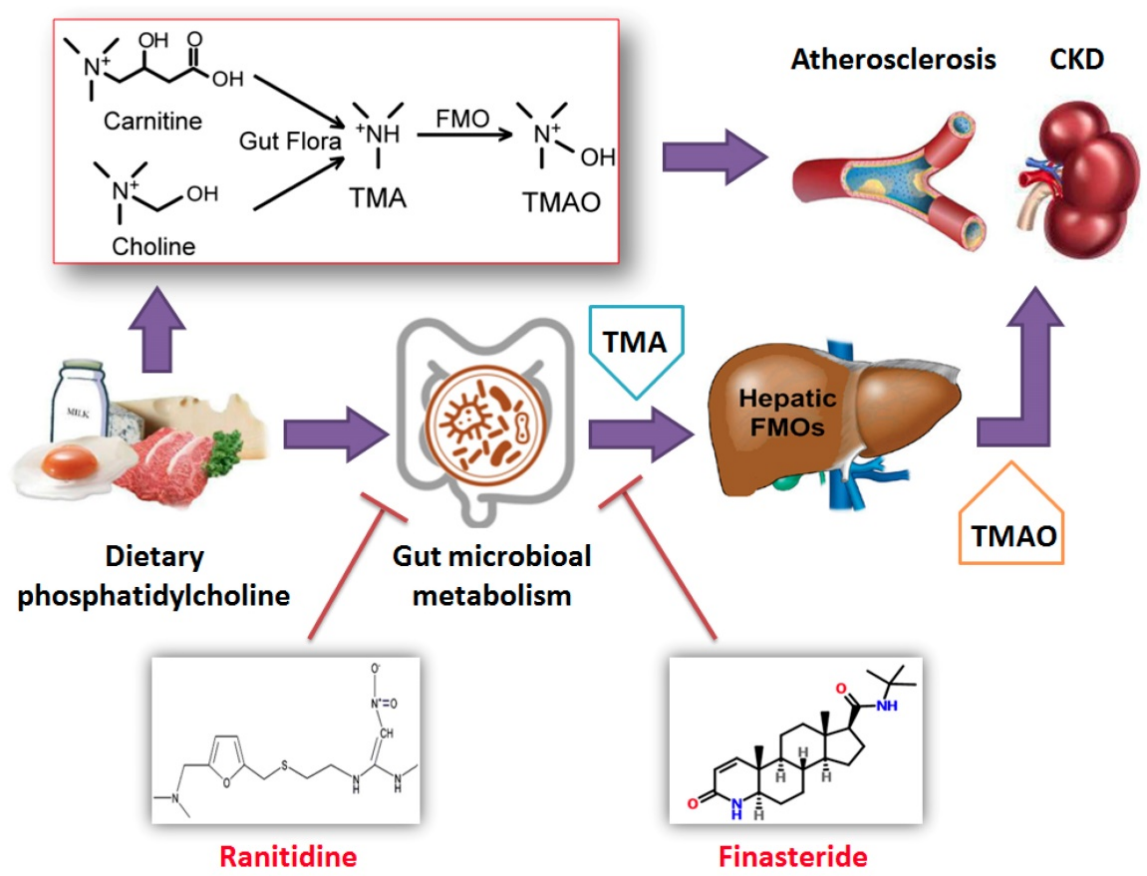
Ranitidine and finasteride inhibit the synthesis and release of trimethylamine N-oxide and mitigates its cardiovascular and renal damage through modulating gut microbiota.

Our study shows that in addition to theirwell-known pharmacological effects, ranitidine and finasteride also exhibit potential cardiovascular and renal protective effects (Figure 8). They can inhibit the synthesis and metabolism of TMAO in the body and delay the deposition of lipids and endotoxins through improving the composition of gut microbiota.

## Supplementary Material

Supplementary figure and tables.
