# Supplementary material for: Ranitidine and finasteride inhibit the synthesis and release of trimethylamine N-oxide and mitigates its cardiovascular and renal damage through modulating gut microbiota
Source: Int J Biol Sci. 2020 Jan 14;16(5):790–802. doi: 10.7150/ijbs.40934 (PMC7019130; doi:10.7150/ijbs.40934)
Supplement: Supplementary file 1 — Supplementary figure and tables. [file ijbsv16p0790s1.pdf]

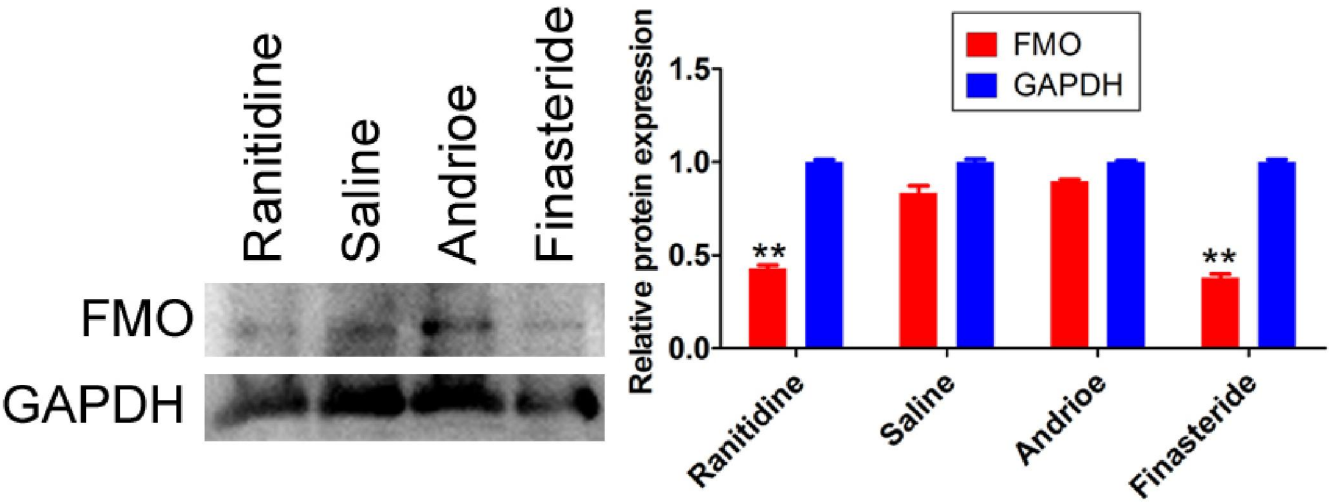

Figure S1 The results of western blot. The result showed that FMO3 expression in the liver tissue of mice treated with ranitidine and finasteride was significantly lower than in the saline-control and Andrioe-treated groups. ** p<0.01 vs Saline group; t test.

Table S1 The results of sample sequencing data processing statistics

Sample ID	PE Reads	Raw Tags	Clean Tags	Effective Tags	AvgLen(bp)	GC(%)	Q20(%)	Q30(%)	Effective(%)
A1	79882	65646	57536	55953	415	54.34	96.61	93.42	70.04
A2	79873	64205	55290	54009	417	55.15	96.67	93.44	67.62
A3	80158	67473	59480	58151	415	54.27	96.68	93.53	72.55
A4	79944	67108	59113	57672	415	53.86	96.48	93.22	72.14
A5	79778	65248	56964	55605	417	54.44	96.58	93.31	69.7
A6	80127	65231	56760	55491	416	54.84	96.64	93.41	69.25
A7	79802	65495	57147	55230	415	54.83	96.59	93.33	69.21
A8	79817	65274	56990	55560	415	54.52	96.63	93.41	69.61
FEN1	80088	67152	59822	56824	415	54.46	96	92.14	70.95
FEN2	79947	67468	59875	57420	418	54.32	96.07	92.29	71.82
FEN3	80259	64784	56786	55039	415	54.7	96.06	92.29	68.58
FEN4	80168	67506	60133	57973	415	54.11	96.11	92.39	72.31
FEN5	7978	6475	5670	54806	415	53.87	96.02	92.21	68.69

	7	1	3						
FEN6	79757	66671	59446	57728	415	54.34	96.17	92.43	72.38
FEN7	80036	67642	60247	56580	416	54.26	96.06	92.31	70.69
FEN8	80363	66476	58623	56923	416	54.37	96.02	92.17	70.83
R1	79880	66720	59074	58359	413	54.24	96.59	93.43	73.06
R2	80209	66801	58929	58311	414	54.29	96.65	93.47	72.7
R3	80125	69146	61848	60935	412	53.73	96.59	93.45	76.05
R4	79974	67256	59662	58542	412	53.82	96.45	93.16	73.2
R5	79681	69802	62870	61949	411	53.44	96.58	93.42	77.75
R6	80302	69030	61638	60710	412	54.25	96.56	93.37	75.6
R7	79786	65186	56758	55797	416	54.41	96.52	93.22	69.93
R8	79781	67524	60009	58603	413	54.27	96.57	93.39	73.45
W1	79973	68241	61037	58773	418	51.61	96.6	93.43	73.49
W2	80317	70466	63381	62267	418	52.17	96.62	93.44	77.53
W3	79824	70045	63173	62099	416	52.08	96.63	93.46	77.79

W4	79779	68859	61547	60488	418	51.68	96.45	93.19	75.82
W5	79921	70445	63563	62460	418	51.59	96.67	93.56	78.15
W6	79760	68691	61725	60874	420	51.06	96.7	93.63	76.32
W7	79721	69411	62435	61246	418	51.79	96.62	93.45	76.83
W8	80360	69787	62511	61299	419	51.51	96.58	93.39	76.28

Table S2 Alpha diversity index statistics

Sample ID	OTU	ACE	Chao1	Simpson	Shannon	Coverage
A1	349	356.2348	357.5714	0.0246	4.4439	0.9996
A2	346	352.2343	356.2	0.0271	4.387	0.9996
A3	359	363.8258	365.5	0.0288	4.471	0.9997
A4	355	361.7728	364.2308	0.0273	4.473	0.9997
R5	354	361.0806	362.5714	0.0339	4.4825	0.9997
R6	338	350.8598	349	0.0419	4.1996	0.9996
R7	349	355.8748	359	0.0195	4.5906	0.9997
R8	349	352.3699	352	0.0508	4.1888	0.9998
W1	54	192.1155	124	0.2482	1.8598	0.9996
W2	40	204.223	79	0.1964	2.0594	0.9998
W3	38	119.3596	71	0.2036	1.9729	0.9998
W4	52	123.5124	103	0.2025	2.1266	0.9997
W5	40	107.9454	58.3333	0.2038	2.0666	0.9998
W6	40	72.5537	49.3333	0.2591	1.8963	0.9999
W7	35	60.8773	45.5	0.2107	1.9424	0.9999
W8	43	111.0345	65.5	0.2583	1.8203	0.9998
A5	359	363.9935	363.1053	0.0228	4.4921	0.9997
A6	363	368.4088	367.5882	0.0208	4.5909	0.9997
A7	353	358.7041	363.9091	0.0225	4.467	0.9996
A8	364	368.4355	370.5	0.024	4.5061	0.9997
FEN1	317	331.6806	332.4762	0.0483	4.0035	0.9992
FEN2	309	327.5397	335.64	0.153	3.0112	0.9991
FEN3	311	320.7279	320.5	0.0384	4.2046	0.9994
FEN4	316	328.1112	328.0476	0.0425	4.0977	0.9994
FEN5	322	329.4535	330.0526	0.0347	4.1957	0.9995
FEN6	322	332.3943	333	0.0571	3.9718	0.9993
FEN7	312	327.3241	329.6522	0.0589	3.7382	0.9992

FEN8	321	330.7174	328.9167	0.0592	3.993	0.9995
R1	351	357.5436	358.5	0.0194	4.6551	0.9997
R2	355	367.3563	367.3529	0.0205	4.612	0.9996
R3	349	356.7963	354.7143	0.0344	4.3975	0.9997
R4	350	354.1829	353.2353	0.0278	4.5348	0.9998

Table S3 Species statistics of different grades of samples

Sample	Kindom	Phylum	Class	Order	Family	Genus	Species
A1	1	10	16	18	32	85	87
A2	1	10	16	17	32	85	85
A3	1	10	16	17	31	83	86
A4	1	10	16	18	33	85	87
A5	1	10	16	17	32	85	87
A6	1	10	16	17	30	84	86
A7	1	10	16	17	30	82	83
A8	1	10	16	17	31	85	87
FEN1	1	10	16	16	29	78	78
FEN2	1	10	16	16	30	77	78
FEN3	1	10	15	15	28	76	77
FEN4	1	10	15	15	26	75	76
FEN5	1	10	16	16	30	80	81
FEN6	1	10	16	16	29	80	81
FEN7	1	10	15	15	29	77	78
FEN8	1	10	16	17	31	80	81
R1	1	10	16	17	31	83	84
R2	1	10	16	17	31	82	83
R3	1	10	16	17	32	82	83
R4	1	10	16	17	30	81	82
R5	1	10	16	17	32	82	83
R6	1	10	16	16	31	80	81
R7	1	10	15	16	31	82	83
R8	1	10	16	17	30	82	84
W1	1	7	11	12	20	39	39
W2	1	6	8	9	16	29	29
W3	1	6	9	10	17	27	27

W4	1	6	8	9	17	39	39
W5	1	7	9	10	16	29	29
W6	1	6	8	9	15	28	28
W7	1	6	8	9	16	28	28
W8	1	8	11	12	19	36	37
Total	1	10	16	19	34	91	94

Table S4 Analysis on the difference of metabolic pathway of KEGG (FEN vs W)

Class1	Class2	FEN	W	FEN: rel. freq. (%)	W: rel. freq. (%)	p-values (corrected)	Effect size	95.0% lower CI	95.0% upper CI
Metabolism	Carbohydrate metabolism	3101849	5511771	14.97520522	15.92648857	0	-0.951283352	-0.970906411	-0.931660293
Metabolism	Lipid metabolism	765678	1434282	3.696564592	4.144416719	0	-0.447852127	-0.458353432	-0.437350821
Metabolism	Metabolism of cofactors and vitamins	1343061	2226544	6.484072597	6.433690292	1.90E-13	0.050382305	0.036985362	0.063779248
Metabolism	Energy metabolism	1429491	2447315	6.901342099	7.071617159	0	-0.170275061	-0.184142747	-0.156407375
Metabolism	Nucleotide metabolism	1117547	1731120	5.395328938	5.002142306	0	0.393186632	0.381037844	0.405335419
Metabolism	Biosynthesis of other secondary metabolites	280970	532746	1.356475899	1.539391437	0	-0.182915538	-0.189376017	-0.176455059
Metabol	Amino acid	2281	3942	11.017	11.392	0	-0.3749	-0.3921	-0.3578

ism	metabolism	995	521	08801	07628		88263	38135	38392
Metabolism	Metabolism of terpenoids and polyketides	388096	696824	1.873662208	2.013501554	0	-0.139839346	-0.147330068	-0.132348624
Metabolism	Xenobiotics biodegradation and metabolism	418000	716849	2.01803369	2.071364614	0	-0.053330924	-0.061031796	-0.045630051
Metabolism	Metabolism of other amino acids	423180	755284	2.043041858	2.182424124	0	-0.139382265	-0.147188053	-0.131576478
Metabolism	Glycan biosynthesis and metabolism	605924	1220926	2.925299152	3.527915798	0	-0.602616647	-0.612134406	-0.593098888
Genetic Information Processing	Translation	1060066	1538206	5.117820338	4.444709383	0	0.673110955	0.66139012	0.68483179
Metabol	Global and	2853	4643	13.777	13.416	0	0.36081	0.34211	0.37950

ism	overview maps	768	194	51188	69967		2207	6202	8213
Human Diseases	Drug resistance	167613	308426	0.80920737	0.891209588	0	-0.082002218	-0.086978721	-0.077025715
Environmental Information Processing	Membrane transport	953344	1468634	4.602584474	4.243678233	0	0.35890624	0.347649834	0.370162647
Environmental Information Processing	Signal transduction	729407	1117801	3.521454305	3.229931877	0	0.291522428	0.281630372	0.301414483
Cellular Processes	Cell motility	396239	257984	1.912975242	0.745455359	0	1.167519883	1.160953959	1.174085808
Genetic Information Processing	Folding, sorting and degradation	446497	757527	2.155612412	2.188905364	2.81E-16	-0.033292952	-0.04123028	-0.025355624
Genetic Information Process	Transcription	46225	69439	0.223166525	0.200646841	0	0.022519684	0.019991741	0.025047626

ing									
Genetic Information Processing	Replication and repair	902255	1423866	4.355935375	4.114319259	0	0.241616116	0.230606005	0.252626227
Organismal Systems	Endocrine system	99947	199848	0.482527304	0.577469	0	-0.094941696	-0.09885805	-0.091025341
Environmental Information Processing	Signaling molecules and interaction	0	0	0	0	1.075	0	-7.56E-06	7.56E-06
Cellular Processes	Cell growth and death	149540	236691	0.721953966	0.683928361	0	0.038025605	0.033453795	0.042597416
Cellular Processes	Transport and catabolism	122564	266873	0.591718376	0.771140489	0	-0.179422113	-0.183834528	-0.175009699
Organismal Systems	Circulatory system	3708	225	0.0179016	0.000650147	0	0.017251454	0.016661517	0.01784139
Organismal System	Development	0	0	0	0	1.048780488	0	-7.56E-06	7.56E-06

s									
Cellular Processes	Cellular community	0	0	0	0	1.023809524	0	-7.56E-06	7.56E-06
Organismal Systems	Immune system	25437	46780	0.122805557	0.13517273	0	-0.012367173	-0.014317193	-0.010417153
Organismal Systems	Environmental adaptation	43076	63132	0.207963682	0.182422506	0	0.025541177	0.023110793	0.027971561
Organismal Systems	Nervous system	68232	135929	0.329412619	0.392772426	0	-0.063359807	-0.0665972	-0.060122413
Organismal Systems	Sensory system	0	0	0	0	1	0	-7.56E-06	7.56E-06
Human Diseases	Endocrine and metabolic diseases	31188	50408	0.150570418	0.145655985	4.70E-06	0.004914433	0.00280861	0.007020256
Organismal Systems	Excretory system	12094	32024	0.058387798	0.092534663	0	-0.034146865	-0.035606465	-0.032687265
Organis	Digestive	4973	1734	0.0240	0.0501	0	-0.0261	-0.0271	-0.0250

mal Systems	system		2	08808	10421		01613	0973	93497
Human Diseases	Neurodegenerative diseases	58696	66457	0.283374415	0.19203023	0	0.091344185	0.088622217	0.094066154
Human Diseases	Substance dependence	9900	23751	0.047795535	0.06862949	0	-0.020833955	-0.022124973	-0.019542937
Human Diseases	Infectious diseases: Bacterial	166745	272220	0.805016812	0.786590865	9.96E-14	0.018425947	0.013573582	0.023278312
Human Diseases	Infectious diseases: Parasitic	11445	26721	0.055254535	0.077211426	0	-0.021956892	-0.023335793	-0.02057799
Human Diseases	Infectious diseases: Viral	20	135	9.66E-05	0.000390088	9.43E-12	-0.000293531	-0.000379329	-0.000207734
Human Diseases	Cancers: Overview	164439	306742	0.793883832	0.886343601	0	-0.092459769	-0.097402682	-0.087516856
Human Diseases	Cancers: Specific types	18513	37798	0.08937765	0.109218873	0	-0.019841223	-0.021542043	-0.018140403
Human Diseases	Immune diseases	11504	23192	0.055539377	0.067014236	0	-0.011474859	-0.012813895	-0.010135823
Human Disease	Cardiovascular	6	45	2.90E-05	0.000130029	7.13E-05	-0.000101062	-0.000153129	-4.90E-05

s	diseases								

Table S5 Analysis on the difference of metabolic pathway of KEGG (R vs W)

Class1	Class2	R	W	R: rel. freq. (%)	W: rel. freq. (%)	p-values (corrected)	Effect size	95.0% lower CI	95.0% upper CI
Metabolism	Carbohydrate metabolism	4869911	5511771	15.71715663	15.92648857	0	-0.209331947	-0.227025874	-0.19163802
Metabolism	Lipid metabolism	1070975	1434282	3.456466005	4.144416719	0	-0.687950714	-0.697201626	-0.678699802
Metabolism	Metabolism of cofactors and vitamins	1933746	2226544	6.240974169	6.433690292	0	-0.192716123	-0.204527452	-0.180904794
Metabolism	Energy metabolism	2012550	2447315	6.495306294	7.071617159	0	-0.576310866	-0.588492336	-0.564129395
Metabolism	Nucleotide metabolism	1742909	1731120	5.625066605	5.002142306	0	0.6229243	0.612029644	0.633818956
Metabolism	Biosynthesis of other secondary metabolites	427351	532746	1.379233132	1.539391437	0	-0.160158305	-0.165968443	-0.154348168
Metabol	Amino acid	3330	3942	10.750	11.392	0	-0.6419	-0.6571	-0.6267

ism	metabolism	889	521	11517	07628		61108	65739	56476
Metabolism	Metabolism of terpenoids and polyketides	523753	696824	1.690361063	2.013501554	0	-0.323140491	-0.329665894	-0.316615088
Metabolism	Xenobiotics biodegradation and metabolism	601689	716849	1.941891803	2.071364614	0	-0.12947281	-0.136270264	-0.122675356
Metabolism	Metabolism of other amino acids	642897	755284	2.074886552	2.182424124	0	-0.107537572	-0.114535481	-0.100539662
Metabolism	Glycan biosynthesis and metabolism	843420	1220926	2.722054724	3.527915798	0	-0.805861075	-0.814269909	-0.79745224
Genetic Information Processing	Translation	1610639	1538206	5.198178248	4.444709383	0	0.753468865	0.743059014	0.763878716
Metabol	Global and	4303	4643	13.888	13.416	0	0.47208	0.45542	0.48874

ism	overview maps	396	194	78543	69967		5758	9782	1734
Human Diseases	Drug resistance	218333	308426	0.704648187	0.891209588	0	-0.186561401	-0.190866108	-0.182256694
Environmental Information Processing	Membrane transport	1509030	1468634	4.870245239	4.243678233	0	0.626567006	0.616434496	0.636699516
Environmental Information Processing	Signal transduction	1066990	1117801	3.443604811	3.229931877	0	0.213672934	0.204953858	0.222392009
Cellular Processes	Cell motility	808152	257984	2.608230738	0.745455359	0	1.862775379	1.856468103	1.869082656
Genetic Information Processing	Folding, sorting and degradation	614923	757527	1.984603231	2.188905364	0	-0.204302134	-0.211227795	-0.197376473
Genetic Information Process	Transcription	68083	69439	0.219731156	0.200646841	0	0.019084315	0.016855496	0.021313134

ing									
Genetic Information Processing	Replication and repair	1374190	1423866	4.435062461	4.114319259	0	0.320743201	0.310922059	0.330564344
Organismal Systems	Endocrine system	143039	199848	0.461644241	0.577469	0	-0.115824759	-0.119304934	-0.112344584
Environmental Information Processing	Signaling molecules and interaction	0	0	0	0	1.075	0	-5.99E-06	5.99E-06
Cellular Processes	Cell growth and death	219593	236691	0.708714713	0.683928361	0	0.024786352	0.020747487	0.028825216
Cellular Processes	Transport and catabolism	174803	266873	0.564159413	0.771140489	0	-0.206981076	-0.210917547	-0.203044606
Organismal Systems	Circulatory system	5082	225	0.016401653	0.000650147	0	0.015751506	0.015286676	0.016216336
Organismal System	Development	0	0	0	0	1.048780488	0	-5.99E-06	5.99E-06

s									
Cellular Processes	Cellular community	0	0	0	0	1.023809524	0	-5.99E-06	5.99E-06
Organismal Systems	Immune system	37758	46780	0.121860215	0.13517273	0	-0.013312516	-0.015052687	-0.011572344
Organismal Systems	Environmental adaptation	69906	63132	0.225614709	0.182422506	0	0.043192203	0.04099257	0.045391836
Organismal Systems	Nervous system	92265	135929	0.297776172	0.392772426	0	-0.094996254	-0.097834824	-0.092157683
Organismal Systems	Sensory system	0	0	0	0	1	0	-5.99E-06	5.99E-06
Human Diseases	Endocrine and metabolic diseases	45984	50408	0.148408817	0.145655985	0.004266266	0.002752832	0.000888971	0.004616692
Organismal Systems	Excretory system	7686	32024	0.024805806	0.092534663	0	-0.067728857	-0.068889691	-0.066568023
Organis	Digestive	7238	1734	0.0233	0.0501	0	-0.0267	-0.0276	-0.0258

mal Systems	system		2	5993	10421		50491	75994	24988
Human Diseases	Neurodegenerative diseases	83772	66457	0.270365854	0.19203023	0	0.078335624	0.075990748	0.0806805
Human Diseases	Substance dependence	3267	23751	0.01054392	0.06862949	0	-0.05808557	-0.05903601	-0.05713513
Human Diseases	Infectious diseases: Bacterial	241479	272220	0.779349615	0.786590865	0.001068603	-0.00724125	-0.011519192	-0.002963307
Human Diseases	Infectious diseases: Parasitic	8287	26721	0.026745474	0.077211426	0	-0.050465953	-0.05156185	-0.049370055
Human Diseases	Infectious diseases: Viral	87	135	0.000280784	0.000390088	0.017815056	-0.000109304	-0.000203679	-1.49E-05
Human Diseases	Cancers: Overview	230224	306742	0.743025215	0.886343601	0	-0.143318386	-0.147671194	-0.138965579
Human Diseases	Cancers: Specific types	23050	37798	0.074391598	0.109218873	0	-0.034827275	-0.036293621	-0.033360929
Human Diseases	Immune diseases	17311	23192	0.055869542	0.067014236	0	-0.011144694	-0.012348871	-0.009940516
Human Disease	Cardiovascular	25	45	8.07E-05	0.000130029	0.056611348	-4.93E-05	-0.000104772	6.08E-06

s	diseases								

Table S6 Analysis on the difference of metabolic pathway of COG (FEN vs W)

Class1	Class2	FEN	W	FEN: rel. freq. (%)	W: rel. freq. (%)	p-values (corrected)	Effect size	95.0% lower CI	95.0% upper CI
INFORMATION STORAGE AND PROCESSING	RNA processing and modification	7950	17702	0.024210278	0.031769065	0	-0.007558787	-0.008272124	-0.006845451
INFORMATION STORAGE AND PROCESSING	Chromatin structure and dynamics	7943	17445	0.024188961	0.031307838	0	-0.007118877	-0.007829791	-0.006407963
METABOLISM	Energy production and conversion	1631110	2685347	4.967248671	4.819283963	0	0.147964708	0.138640832	0.157288584
CELLULAR PROCESSES AND SIGNALING	Cell cycle control, cell division, chromosome partitioning	385875	544647	1.17511209	0.977456006	0	0.197656084	0.19315042	0.202161748
METABOLISM	Amino acid transport and	243776	398004	7.423775766	7.14281907	0	0.280956696	0.269721392	0.292191999

	metabolism	7	1						
METABOLISM	Nucleotide transport and metabolism	834995	1254413	2.542825318	2.251244422	0	0.291580896	0.284930729	0.298231063
METABOLISM	Carbohydrate transport and metabolism	2818152	4502184	8.58216906	8.079888055	0	0.502281005	0.490318641	0.514243369
METABOLISM	Coenzyme transport and metabolism	1302304	2300565	3.965929835	4.128731225	0	-0.162801391	-0.171282219	-0.154320563
METABOLISM	Lipid transport and metabolism	800689	1454459	2.438352638	2.61025891	0	-0.171906272	-0.178645637	-0.165166908
INFORMATION STORAGE AND PROCESSING	Translation, ribosomal structure and biogenesis	1942656	2911433	5.916005317	5.225031389	0	0.690973928	0.681006575	0.700941282
INFORMATION STORAGE AND PROCESSING	Transcription	2618402	4231142	7.973866786	7.593459908	0	0.380406878	0.368816852	0.391996904
INFORMATION STORAGE AND	Replication, recombination and repair	2016463	3358575	6.140771104	6.027499104	0	0.113272001	0.102948548	0.123595454

PROCESSING									
CELLULAR PROCESSES AND SIGNALING	Cell wall/membrane/envelope biogenesis	2382023	4532924	7.254017559	8.135055893	0	-0.881038335	-0.892454763	-0.869621906
CELLULAR PROCESSES AND SIGNALING	Cell motility	615110	772156	1.873205565	1.385757233	0	0.487448333	0.481882626	0.493014039
CELLULAR PROCESSES AND SIGNALING	Posttranslational modification, protein turnover, chaperones	1058478	1819086	3.223402124	3.264640282	0	-0.041238158	-0.048876077	-0.033600239
METABOLISM	Inorganic ion transport and metabolism	1555870	3178716	4.738118943	5.70471341	0	-0.966594467	-0.976080125	-0.957108808
METABOLISM	Secondary metabolites biosynthesis, transport and catabolism	383771	744720	1.168704741	1.33651895	0	-0.167814209	-0.172573237	-0.163055181
POORLY	General	383	675	11.684	12.115	0	-0.431	-0.445	-0.417

CHARACTERIZED	function prediction only	6868	1015	48325	77436		291108	228777	353439
POORLY CHARACTERIZED	Function unknown	2457564	4518638	7.484063927	8.109417385	0	-0.625353458	-0.636863551	-0.613843365
CELLULAR PROCESSES AND SIGNALING	Signal transduction mechanisms	2038226	3242537	6.20704636	5.819250385	0	0.387795976	0.377500958	0.398090993
CELLULAR PROCESSES AND SIGNALING	Intracellular trafficking, secretion, and vesicular transport	791901	1487278	2.411590383	2.669157846	0	-0.257567463	-0.264313263	-0.250821662
CELLULAR PROCESSES AND SIGNALING	Defense mechanisms	907769	1404743	2.764445291	2.52103561	0	0.243409681	0.236448798	0.250370564
CELLULAR PROCESSES AND SIGNALI	Extracellular structures	16	735	4.87E-05	0.001319075	1.13E-122	-0.00127035	-0.001373397	-0.001167302

NG									
CELLULAR PROCESSES AND SIGNALING	Nuclear structure	0	0	0	0	1	0	-4.74E-06	4.74E-06
CELLULAR PROCESSES AND SIGNALING	Cytoskeleton	5391	10370	0.016417309	0.018610621	5.87E-14	-0.002193311	-0.00276401	-0.001622613

Table S7 Analysis on the difference of metabolic pathway of COG (R vs W)

Class1	Class2	R	W1	R: rel. freq. (%)	W: rel. freq. (%)	p-values (corrected)	Effect size	95.0% lower CI	95.0% upper CI
INFORMATION STORAGE AND PROCESSING	RNA processing and modification	6762	17702	0.013342264	0.031769065	0	-0.018426801	-0.018996238	-0.017857365
INFORMATION STORAGE AND PROCESSING	Chromatin structure and dynamics	4840	17445	0.00954992	0.031307838	0	-0.021757918	-0.022298407	-0.021217429
METABOLISM	Energy production and conversion	2455957	2685347	4.845907602	4.819283963	1.74E-10	0.026623639	0.018460659	0.034786619
CELLULAR PROCESSES AND SIGNALING	Cell cycle control, cell division, chromosome partitioning	661520	544647	1.305260962	0.977456006	0	0.327804956	0.32374699	0.331862923
METABOLISM	Amino acid transport and	374466	398004	7.388684288	7.1428 1907	0	0.245865218	0.235982664	0.255747771

	metabolism	3	1						
METABOLISM	Nucleotide transport and metabolism	1379629	1254413	2.722179036	2.251244422	0	0.470934615	0.464994384	0.476874845
METABOLISM	Carbohydrate transport and metabolism	5389660	4502184	10.63446728	8.079888055	0	2.554579228	2.543474335	2.565684121
METABOLISM	Coenzyme transport and metabolism	1879108	2300565	3.707713019	4.128731225	0	-0.421018207	-0.428394145	-0.413642269
METABOLISM	Lipid transport and metabolism	1092155	1454459	2.154957199	2.61025891	0	-0.455301711	-0.461093976	-0.449509446
INFORMATION STORAGE AND PROCESSING	Translation, ribosomal structure and biogenesis	2988494	2911433	5.896669116	5.225031389	0	0.671637727	0.662904824	0.68037063
INFORMATION STORAGE AND PROCESSING	Transcription	4580517	4231142	9.037927843	7.593459908	0	1.444467934	1.433943399	1.454992469
INFORMATION STORAGE AND	Replication, recombination and repair	3065461	3358575	6.048534548	6.027499104	5.59E-06	0.021035444	0.011969606	0.030101282

PROCESSING									
CELLULAR PROCESSES AND SIGNALING	Cell wall/membrane/envelope biogenesis	3269816	4532924	6.451752295	8.135055893	0	-1.683303598	-1.693169778	-1.673437419
CELLULAR PROCESSES AND SIGNALING	Cell motility	953023	772156	1.880432516	1.385757233	0	0.494675283	0.489833593	0.499516973
CELLULAR PROCESSES AND SIGNALING	Posttranslational modification, protein turnover, chaperones	1472292	1819086	2.905014622	3.264640282	0	-0.35962566	-0.366198328	-0.353052993
METABOLISM	Inorganic ion transport and metabolism	2181212	3178716	4.303801659	5.70471341	0	-1.40091175	-1.409179992	-1.392643508
METABOLISM	Secondary metabolites biosynthesis, transport and catabolism	482003	744720	0.951051668	1.33651895	0	-0.385467282	-0.389499754	-0.38143481
POORLY	General	566	675	11.168	12.115	0	-0.947	-0.959	-0.935

CHARAC TERIZED	function prediction only	025 5	101 5	38476	77436		389596	583689	195503
POORLY CHARAC TERIZED	Function unknown	340 948 7	451 863 8	6.7273 40492	8.1094 17385	0	-1.382 076894	-1.392 027152	-1.372 126635
CELLULA R PROCES SES AND SIGNALI NG	Signal transduction mechanisms	339 624 0	324 253 7	6.7012 02518	5.8192 50385	0	0.8819 52134	0.8727 19511	0.8911 84757
CELLULA R PROCES SES AND SIGNALI NG	Intracellular trafficking, secretion, and vesicular transport	978 971	148 727 8	1.9316 31136	2.6691 57846	0	-0.737 526709	-0.743 21095	-0.731 842469
CELLULA R PROCES SES AND SIGNALI NG	Defense mechanisms	162 184 2	140 474 3	3.2000 9531	2.5210 3561	0	0.6790 597	0.6726 98208	0.6854 21192
CELLULA R PROCES SES AND SIGNALI	Extracellular structures	7	735	1.38E-05	0.0013 19075	5.98E -193	-0.001 305263	-0.001 404864	-0.001 205662

NG									
CELLULAR PROCESSES AND SIGNALING	Nuclear structure	0	0	0	0	1	0	-3.69E-06	3.69E-06
CELLULAR PROCESSES AND SIGNALING	Cytoskeleton	7139	10370	0.014086132	0.018610621	0	-0.004524489	-0.005012983	-0.004035994
